# 

**Published:** 1912-09-07

**Authors:** 


### BUREAU OF INFORMATION.

### Rules for Correspondents.

1. Every letter, on whatever subject, **must be accompanied by the coupon** to be cut from the back cover (inside page) of The Hospital, current issue, and must contain the name and address of the correspondent with pseudonym for publication if desired.

2. Replies by post cannot be given, save under exceptional circumstances at the Editor's discretion.

3. Letters from Approved Homes in reply to special needs published in the Bureau should state terms and full particulars, and be sent prepaid under cover to the Editor of the Bureau with coupon and name enclosed for identification.

4. Proprietors of Homes which have not yet been entered on the List of Approved Homes, but have spare accommodation likely to suit special needs, are invited to write for an application form for registration. The fee for registration, which includes two announcements of the Home in the Bureau, is 5s.

5. Special needs of every description relating to those who are sick or in difficulties are made known in these columns **free of charge.**

6. All communications to be addressed to The Editor of The Hospital, 28 Southampton Street, Strand, London, W.C., and marked "Bureau of Information."

### Needs and Helpers.

**Home Wanted near Cardiff.**—Can our Cardiff correspondent kindly inform us whether there is any home in the vicinity where a person with creeping paralysis could be received who can afford to pay 15s. a week? Prepaid letters forwarded to Sister E. M. (120z).

**Home for Delicate Boy.**—The Editor regrets that the boy recovering from nephritis is considered too much of an invalid for the home recommended. It appears that the home, which has no trained matron, has had a bad failure with a similar case, and fears the responsibility. Can any one of our readers help F. M. D. to provide for this case, which comes strongly supported in every direction except money?

**Home of Rest for Gentlewomen.**—We have forwarded on letter, and shall be much obliged if you will send us in return a copy of the rules of admission to the S. P. G. Home at Worthing.—Mrs. L.

### Training and Employment.

**Too Old at Forty.**—It is useless to try to obtain employment on one of the best nursing co-operations, not because you are too old for nursing, but because all such institutes have already as many nurses as they require at that age, and prefer to recruit the ranks with nurses fresh from hospital. You will find innumerable district posts advertised where very young women are not desired, and with the qualifications you name it is only a question of perseverance before you succeed in getting employment in any line you may select, except the one we have named. Please read over our rules and notice which you have broken.—M.

**Children's Sanatoriums.**—The only way to procure the situation you require is to have your testimonials typed, and to send them with a letter stating your training and previous experience in reply to any announcement in the nursing and medical papers of nurses wanted in institutions of this nature. We must warn you that there are very few sanatoriums for children, and that vacancies rarely occur.—G. G. S. (Knutsford).

### Approved Homes.

Note.—*No Home is added to the List without direct medical and other testimony to its good management.*

**Nursing Home at St. Leonards-on-Sea.**—87 Marina St. Leonards-on-Sea. Superintendent, Miss Greenwood. Medical, surgical, slight mental, chronic, and convalescent cases. Very kind care taken of the aged and helpless.

**High-Class Nursing Home in London.**—5 Highbury Quadrant, London, N. Proprietors: Miss Evelyne Johns, Miss Florence Johns. Certificated, trained nurses. Medical, surgical, maternity, slightly mental, nerve cases. Well situated on high ground in quiet situation. Garden. Patients visited by their own doctors.

### TRAINING IN SWEDISH MASSAGE.

One of the constant questions which is sent to our Bureau of Information is "How and where can I be trained in Swedish massage?" There still seems, in short, a good deal of ignorance on the subject of existing training schools. For this reason the prospectus of the Swedish Institute and Clinique, 106 and 108 Cromwell Road, S.W., is of interest. The school was founded in 1904, its principal is Mrs. Coghill-Hawkes, M.D., and its aim is to train educated gentlewomen in the same principles as those of the Swedish training schools. In addition to theoretical instruction, practical work is given in connection with the London hospitals. The fee for one year's training is £63. The autumn session starts this month. It is also satisfactory to note the statement in the prospectus to the effect that "no difficulty has been experienced in finding work for the students."

